# Oral Cavity as an Extragastric Reservoir of *Helicobacter pylori*

**DOI:** 10.1155/2014/261369

**Published:** 2014-02-20

**Authors:** Arwa Al Sayed, Pradeep S. Anand, Kavitha P. Kamath, Shankargouda Patil, R. S. Preethanath, Sukumaran Anil

**Affiliations:** ^1^Department of Dentistry, Prince Sultan Military Medical City, Ministry of Defense, P.O. Box 2993, Riyadh 11461, Saudi Arabia; ^2^Department of Periodontics, People's College of Dental Sciences & Research Centre, Bhopal, Madhya Pradesh 462037, India; ^3^Department of Oral Pathology, People's Dental Academy, Bhopal, Madhya Pradesh 462037, India; ^4^Department of Oral Pathology, MS Ramaiah Dental College and Hospital, Bangalore, Karnataka 560054, India; ^5^Department of Periodontics and Community Dentistry, College of Dentistry, King Saud University, P.O. Box 60169, Riyadh 11545, Saudi Arabia

## Abstract

*Background*. Several studies were reported on the prevalence, and relationship between the existence of *Helicobacter pylori* (*H. pylori*) in oral cavity and in stomach of patients. The purpose of this study was to systematically review the existing literature on the presence of *H. pylori* in the oral cavity and its link to gastric infection, the existence of coinfection, and the impact of anti-*H. pylori* therapy on the dental plaque and vice versa. *Method*. Two authors independently searched the Medline, EMBASE, Cochrane Library, Web of Science, Google Scholar, and Scopus databases for relevant studies. The articles were analyzed critically and all qualified studies were included. The search was carried out by using a combined text and the MeSH search strategies: using the key words *Helicobacter*, *Helicobacter pylori*, and *H. pylori* in combination with dental plaque, periodontitis, and oral hygiene. *Results*. The data was presented in 8 tables and each topic separately discussed. *Conclusion*. Based on the systematic review of the available literature on *H. pylori* infection and its presence in the oral cavity, it can be concluded that dental plaque can act as a reservoir, and proper oral hygiene maintenance is essential to prevent reinfection. Due to the diversified methods and population groups involved in the available literature, no concrete evidence can be laid down. Further studies are necessary to establish the role of *H. pylori* in the oral cavity and its eradication on preventing the gastroduodenal infection.

## 1. Introduction

*Helicobacter pylori* (*H. pylori*) is one of the most common bacterial infections in humans [1]. The presence of the organism *H. pylori* (initially termed* Campylobacter pyloridis*) in the antral mucosa of humans was first reported in 1983 [2].* H. pylori* has been closely linked to chronic gastritis, peptic ulcer, gastric cancer, and mucosa-associated lymphoid tissue (MALT) lymphoma [3, 4]. The International Agency for Research on Cancer of the World Health Organization (WHO) has designated *H. pylori* as a Group 1 carcinogen [5]. Besides gastrointestinal diseases, recent data seems to suggest a possible association of this microorganism with other conditions such as anemia [6], altered serum levels of lipoproteins [7], and coronary atherosclerosis [8]. Although *H. pylori *is present in the stomach of about half of the world's population, we do not yet clearly understand its transmission. Available data suggests that oral-oral and fecal-oral routes are the most likely routes of transmission of this organism [9, 10]. However, no extragastric reservoirs of *H. pylori *have been clearly demonstrated. A recent study has reported the detection of the organisms in soil samples in public playgrounds suggesting the role of the abovementioned routes in the transmission of the organism [11]. However, the likelihood of transmission of infection through contaminated soil needs to be clarified. Although organisms resembling *H. pylori *may be detected in other animals, none, except non-human primates [12] and cats [13], harbor *H. pylori*. Infections by *Helicobacter* species (*H. heilmannii *and* H. felis*) have been reported in dogs and cats [14, 15]. It has been suggested that the microorganism may be transmitted orally and has been detected in dental plaque and saliva [16–18]. But whether the oral cavity serves as an extragastric reservoir for *H. pylori *or harbors the organism only transiently, is not yet clear (the organism being only a transient inhabitant of this ecological niche or not). If the oral cavity is an extragastric reservoir of the *H. pylori*, it may be clinically significant from the treatment aspect as the microorganisms residing in the dental plaque are afforded protection from systemically administered antimicrobial agents. Treatment of *H. pylori *infection usually involves a combination of antibiotics, acid suppressors, and stomach protectors. Despite the current treatment regimens that lead to successful management of *H. pylori*—positive chronic gastritis, the reinfection rate is relatively high [19]. One of the suggested mechanisms of reinfection is the possible recolonization from the dental plaque [20]. A large number of studies have been carried out among various populations to determine whether dental plaque and periodontal disease are associated with *H. pylori* infection. This paper attempts to review the current evidence regarding the role of oral cavity as an extragastric reservoir of *H. pylori*.

## 2. Materials and Methods

### 2.1. Literature Search

A systematic review was conducted in January 2013. All relevant studies published between January 1990 and December 2012 were identified and included in the systematic analysis. Two authors independently searched the Medline, EMBASE, Cochrane Library, Web of Science, Google Scholar, and Scopus databases for relevant studies. The search was carried out by using a combined text and the MeSH search strategies: using the key words *Helicobacter*, *Helicobacter pylori*, and *H. pylori* in combination with dental plaque, periodontitis, and oral hygiene. We also examined the bibliographies from identified studies, reviews, and gray literature. The last search was conducted on December 31, 2012.

### 2.2. Study Selection Criteria

Studies reporting the identification of *Helicobacter pylori* in dental plaque, coinfection of periodontitis and *H. pylori,* effect of periodontal therapy on *H. pylori, *and effect of treatment of* H. pylori *infection on periodontal problems were included in the review. The types of studies included were cross-sectional, experimental studies and interventional studies. Patients in all age groups were included. Studies presented solely in the form of abstracts in scientific conferences and studies published in languages other than English were not considered in this review.

### 2.3. Data Extraction Considerations

Data extracted from each of the included studies was referred to the study design, the method used to study the presence of *H. pylori, *and the type of association between the periodontal problems and *H. pylori* infection. The data was presented in a tabular form with the variables in quantitative and qualitative format.

The papers were grouped according to the content of the study and presented in 8 tables based on the date of publication of the study. Tables 1–4 depict the presence of *H. pylori *in dental plaque. Tables 5 and 6 enumerate the coinfection and association studies between *H. pylori* infection and periodontal disease.  Table 7 shows the effect of anti-*H. pylori* therapy on its presence in dental plaque. Studies on the effects of periodontal treatment on *H. pylori* presence in dental plaque and gastric infection are listed are Table 8.

### 2.4. Methods of Detection of *H. pylori*

Various methods have been employed to detect the presence of the bacterium *H. pylori* in the gastrointestinal mucosa. These include histology, culture, urease test, serologic tests, urea breath test, and polymerase chain reaction targeting specific nuclear material of the microorganism [3]. Histological methods using conventional hematoxylin and eosin staining can be used to visualize *H. pylori* while use of special stains such Warthin-Starry and Giemsa staining can enhance the histologic identification of the microorganism. By employing culture methods, antimicrobial susceptibility tests can be performed. Urease tests and urea breath tests are based on the fact that the microorganisms are associated with large amounts of urease activity while serologic tests detect the levels of antibodies such as IgG and IgA in the serum that is elevated in response to an infection by *H. pylori*. Several different polymerase chain reaction (PCR) methods which differ in their target DNA have been developed for the diagnosis of *H. pylori* infection and these can help to differentiate between *H. pylori* strains.

### 2.5. *H. pylori* in Dental Plaque

The prevalence of *H. pylori* in the dental plaque has been studied by several investigators. The results of these studies showed wide variation and this seems to depend at least in part on the method employed to detect the bacterium in the dental plaque. As mentioned earlier, investigators have used several methods to detect the presence of the bacterium in the dental plaque and these include urease tests (rapid urease/CLO test), PCR, histology, culture, and immunoassays.

### 2.6. Prevalence Data as Reported in Studies Utilizing Urease Tests

The prevalence of *H. Pylori* in the dental plaque of study participants reported by investigators using urease tests is given in Table 1. The prevalence of *H. pylori* in dental plaque in these studies generally ranged from 50% to 100% except in 3 studies. In 2 of these studies the prevalence rates reported were 44.8% [21] and 43% [22] while the lowest rate reported was 18.2% [23].

### 2.7. Prevalence Data as Reported in Studies Utilizing PCR

The prevalence of *H. pylori* in the dental plaque of study participants reported by investigators using PCR is given in Table 2. The prevalence rates reported in these studies ranged from 0-100% and were generally lower than those reported in studies in which urease tests were used to detect the presence of *H. pylori* in dental plaque. However, out of the 34 studies reviewed in this category, only 7 studies reported a prevalence rate exceeding 50%. Of these 7 studies, 6 were conducted amongst Asian populations.

### 2.8. Prevalence Data as Reported in Studies Utilizing Culture

The prevalence of *H. pylori* in the dental plaque reported by investigators using culture method is given in Table 3. The prevalence rates reported in these studies were generally below 50% with about half studies reported less than 10% prevalence. In 2 of these studies [24, 25], the microorganism could not be cultured from the dental plaque.

### 2.9. Prevalence Data as Reported in Studies Utilizing Immunoassays

The prevalence of *H. pylori* in the dental plaque reported by investigators using immunoassays is given in Table 4. Using this method for detection of *H. pylori* in dental plaque, 2 studies [26, 27] reported a high prevalence (>65%) of the microorganism in dental plaque while other 2 studies [28, 29] reported a very low prevalence (0 and 11%).

### 2.10. Prevalence of Coinfection of Gastric Mucosa and Dental Plaque by *H. pylori*

The summary of studies which have evaluated the prevalence of coinfection of gastric mucosa and dental plaque by *H. pylori* among the study participants is given in Table 5. The prevalence rate of coinfection among the respective study populations reported by different investigators ranged from as low as 1% to as high as 82.1%. This wide variation in the prevalence rates of coinfection may be partly due to the difference in the diagnostic tests employed by various investigators to detect the bacterium in the dental plaque. Studies utilizing urease tests to detect the presence of the microorganism in the dental plaque have reported very high prevalence rates. Except for one study which reported a 25.2% prevalence of coinfection, all the other studies utilizing urease tests have reported a coinfection rate in excess of 32%, with one study reporting a prevalence rate of 81.3% [30]. In studies involving PCR, the prevalence rates have ranged between 0% and 47.6% with only 5 out of the 11 studies reporting a prevalence rate above 30%. Low rates of prevalence of coinfection were reported when microbial culture was employed to detect *H. pylori* from the dental plaque. Out of the 5 studies reviewed which employed microbial culture, the rates of prevalence reported were 1%, 1.4%, 6.9%, 14.6%, and 18%, respectively [16, 31–33]. In the 2 studies reviewed which used immunoassays for detection of pathogen in dental plaque samples, the prevalence rates reported were 23.6% and 47.6% [27]. Among patients with gastrointestinal colonization by *H. pylori*, the prevalence of coinfection in dental plaque was reported to be in the range of 25.2% to 100%. In studies involving the use of urease test only 2 out of the 8 studies reported a prevalence rate of less than 50%; 0% to 100% in PCR studies with 5 out of the 11 studies reviewed reported a prevalence rate above 50%; 1.7% to 30% in the 5 studies using microbial culture; and 23.6% to 82.1% in studies using immunoassays.

### 2.11. *H. pylori* and Periodontal Disease

Few studies have evaluated the relationship between gingival and periodontal disease and *H. pylori* infection. While some investigators [34, 35] have reported a positive association between the two conditions, others have reported that there was no association between *H. pylori* infection and periodontal diseases [18, 36].  Table 6 shows the studies which have evaluated the relationship between periodontal disease and *H. pylori* infection.

A large-scale epidemiological study which evaluated the relationship between *H. pylori* infection and abnormal periodontal conditions by Dye et al. [34] utilized the data from the first phase of the third National Health and Nutrition Examination Survey. A total of 4504 participants aged 20 to 59 years who completed a periodontal examination and tested positive for *H. pylori* antibodies were examined. Periodontal pockets with a depth of 5 mm or more were associated with increased odds of *H. pylori *seropositivity after adjustment for sociodemographic factors. The authors reported that this association is comparable to the independent effects of poverty on *H. pylori* and concluded that poor periodontal health, characterized by advanced periodontal pockets, may be associated with *H. pylori* infection in adults, independent of poverty status.

Nested polymerase chain reaction (PCR) was employed by Umeda et al. [37] to clarify whether the oral cavity acts as a reservoir for *H. pylori*. The existence of *H. pylori* in the oral cavity was determined by nested PCR in 57 subjects and by culture method in 18 subjects. The presence of periodontopathic bacteria was also determined by 16S rRNA-based PCR method. Although *H. pylori* was rarely detected in the oral cavity by culture technique, it was frequently detected (35.1%) by nested PCR, especially among periodontitis patients who had the bacterium in the gastrointestinal tract (46.4%). Among the subjects who harbored *H. pylori* in the stomach or duodenum, 41.2% of patients with periodontal pockets ≥4 mm and 9.1% of subjects without periodontal pockets showed *H. pylori* in dental plaque. They also reported that one patient who had periodontal pockets retained *H. pylori* in the oral cavity even after eradication of the bacterium from the stomach and duodenum. Most (8/10) of the patients who had *H. pylori* in dental plaque harbored *Bacteroides forsythus* in their oral cavities. Based on the previously mentioned findings, the authors concluded that close attention should be given to periodontitis patients who harbor *H. pylori* in the oral cavity.

Association between periodontal disease and *H. pylori* infection was tested in a case-control study among 134 dyspeptic patients reporting for upper gastrointestinal endoscopy [38]. The periodontal status of the patients was examined as a dichotomous variable with patients being described as being either diseased or healthy depending on their periodontal status. Among the cases, 30 subjects out of 65 (46.2%) had periodontal disease compared to only 20 out of 69 (29%) in comparison to the controls. Although the univariate analysis suggested that the relationship was significant, when analyzed by logistic regression, the difference was found to be not significant.

Lack of association between *H. pylori* infection and periodontal disease was reported by Berroteran et al. [39] based on the results from their study of a Venezuelan population. Gingival and Plaque indices were used to assess the gingival and oral hygiene status of the 32 dyspeptic patients and 20 asymptomatic subjects. It was found that patients with poor oral hygiene and periodontal status had a similar prevalence of *H. pylori* to patients with good-to-moderate dental hygiene.

To elucidate the possible sources of *H. pylori* infection in an isolated, rural population in Guatemala, Dowsett et al. [18] examined 242 subjects in family units. Periodontal status, *H. pylori* antibody status, and presence of *H. pylori* in the dental plaque, dorsum of tongue, and fingernails were recorded. PCR based on 16S rRNA gene of *H. pylori* were employed for the detection of the microorganism in the plaque, tongue, and finger nail samples. It was found that there was no statistically significant relationship between *H. pylori* status and periodontal disease. A high rate of oral carriage was found irrespective of periodontal status, showing no association with pocket depth.

Al Asqah et al. [35] reported that 60% (37/62) of the periodontitis patients in their study harbored *H. pylori* in their stomach compared to only 33% (13/39) of the patients without periodontitis. Furthermore, they reported that the prevalence of *H. pylori* in the dental plaque was higher among periodontitis patients (79%, 49/62) than in patients without periodontitis (43%, 17/39). They also reported that the presence of the bacterium in both locations was higher among periodontitis patients (46.8%, 29/62) than in patients without periodontitis (10.3%, 4/39).

### 2.12. Effects of Systemic Anti-*H. pylori* Therapy on Dental Plaque

A total of 8 studies [30, 40–46] were reviewed in which the effect of anti-*H. pylori* therapy on its presence in the dental plaque was evaluated. The summary of these studies are given in Table 7. In 6 of these 8 studies, PCR was used to detect *H. pylori* in dental plaque while in one study [30] urease test was used and in the other [46] smear cytology was used. In their study on 82 *H. pylori* positive patients, Butt et al. [46] treated 27 of these patients with anti-*H. pylori* therapy (triple therapy-2 antibiotics and 1 proton pump inhibitor) alone and reported 100% prevalence of *H. pylori* in the dental plaque of these 27 patients after 10 days of treatment. Gürbüz et al. [30] conducted a study among 75 dyspeptic patients in which *H. pylori* positive patients were treated with anti-*H. pylori* therapy. In this study, initially 68 (91%) patients were positive for *H. pylori* in dental plaque and 65 (87%) were positive for *H. pylori* in the gastric mucosa. When the procedures were repeated after 1 month following treatment, the authors reported that all the dental plaque samples were positive for *H. pylori* although they had not mentioned the number of patients treated with anti-*H. pylori* therapy. Among the studies in which PCR was employed for pathogen detection, Gao et al. [44] reported that, among 37 *H. pylori* positive patients, the prevalence of plaque colonization was 29.7% and 43.2%, respectively, at 4 weeks and 1 year after anti-*H. pylori* therapy. Gebara et al. [42], in their study on 30 dental patients with periodontitis and *H. pylori* infection, reported an increase in the prevalence of plaque colonization from 20% to 30% in supragingival plaque and from 26.6% to 46.7% in subgingival plaque. Suk et al. [45] reported that, after anti-*H. pylori* therapy, the microorganism persisted in the dental plaque of 92.9% (*n* = 26) of the 28 patients who harbored the organism in the dental plaque before anti-*H. pylori* therapy. Zaric et al. [40] reported that, after anti-*H. pylori *therapy, the pathogen could be detected in the dental plaque of 66.7% (*n* = 14) of the 21 patients who were positive for the microorganism both in the subgingival plaque and gastric mucosa before the intervention. Miyabayashi et al. [43] in their study on 47 dyspeptic patients reported 48.9% (*n* = 23) positive for oral *H. pylori* and 38.3% (*n* = 18) had *H. pylori* in plaque before anti-*H. pylori* treatment. At 4 weeks after treatment, they reported that 31.9% of the patients were positive for oral *H. pylori*. However, they did not specify how many patients were positive for the microorganism in the dental plaque. Contrary to these studies, Bago et al. [41], reported that 21 patients were positive for *H. pylori* in the dental plaque in a study on 56 chronic periodontitis patients who harbored *H. pylori* in the gastric mucosa. They reported complete eradication of *H. pylori* from dental plaque in all of the 21 patients following anti-*H. pylori* therapy consisting of 2 antibiotic and 1 proton pump inhibitor (PPI).

### 2.13. Effects of Periodontal Therapy on Dental Plaque-Associated *H. pylori*

Three studies have evaluated the effects of nonsurgical periodontal therapy on *H. pylori* residing in the dental plaque (Table 8). Butt et al. [46] categorized 82 patients who harbored *H. pylori* in their dental plaque into 3 groups based on the type of intervention—Group 1 which received only anti-*H. pylori* therapy (*n* = 27), Group 2 which received anti-*H. pylori* therapy plus periodontal therapy (*n* = 25), and Group 3 which received only periodontal therapy (*n* = 30). Ten days after treatment, the prevalence of *H. pylori* in dental plaque for Groups 1, 2, and 3 were 100%, 16%, and 10%, respectively. In a study on 43 patients who harbored *H. pylori* both in the subgingival plaque and gastric mucosa, Zaric et al. [40] reported that, among 22 patients who received both anti-*H. pylori* therapy and periodontal therapy, *H. pylori* was detected in the dental plaque of only 6 patients 3 months after completion of treatment compared to 21 patients who received only anti-*H. pylori* therapy among whom the prevalence after intervention was 66.7%. Gao et al. [44], in their study to evaluate the effects of combination of anti-*H. pylori* therapy (triple therapy) and periodontal therapy for the management of *H. pylori* infection, treated 37 patients with anti-*H. pylori* therapy alone and 43 patients with a combination of anti-*H. pylori* therapy and periodontal therapy. The detection rates of *H. pylori* in the dental plaque for both groups at 4 weeks after intervention were 29.7% and 4.7%, respectively, and 43.2% and 18.6%, respectively, one year after intervention.

### 2.14. Effects of Periodontal Therapy on Gastric *H. pylori* Infection

Three studies have evaluated the effects of periodontal therapy on gastric *H. pylori* infection. Gao et al. [44] reported that, among 43 *H. pylori* positive patients who received both anti-*H. pylori* therapy and periodontal therapy, the gastric eradication rate at 4 weeks and 1 year after intervention was 81.4% and 62.8%, respectively, while the eradication rates at same time periods among 37 *H. pylori* positive patients who received only anti-*H. pylori* therapy were 73% and 32.4%. Zaric et al. [40] conducted a study among 43 patients who were positive for *H. pylori* in both subgingival plaque and gastric mucosa in which 21 patients received only anti-*H. pylori* therapy while 22 received anti-*H. pylori* therapy along with periodontal therapy. Three months after completion of treatment, 77.3% of the patients who received both anti-*H. pylori* therapy and periodontal therapy showed gastric eradication compared to only 47.6% of the patients who received only anti-*H. pylori* therapy. The authors also reported that eradication in the stomach coincided with eradication from the oral cavity; that is, all 16 of the individuals who received both forms of therapy and showed eradication of oral *H. pylori*, also showed eradication of gastric *H. pylori*. Five of the participants in this group who were positive for oral samples were positive for gastric *H. pylori *as well. Jia et al. [47], in a study on 107 *H. pylori *positive dyspeptic patient, reported that, 6 months after complete eradication of *H. pylori* from gastric mucosa, reinfection of the gastric mucosa by the bacterium was observed in 84.31% of the patients who did not receive any form of dental plaque control compared to only 19.64% of the patients who received dental plaque control and full-mouth scaling and root planing. 

## 3. Conclusion

*H. pylori *is a major etiologic factor in the development of gastritis and peptic ulcer disease. There is sufficient evidence on the presence of *H. pylori* in the subgingival oral biofilm which could act as a reservoir for harboring *H. pylori*, leading to gastric reinfection. Hence, it is imperative to adapt a multidisciplinary clinical management protocol, merging the triple therapy to periodontal mechanical treatment and chemical antiseptic disinfection. Further research that may be directed towards controlled randomized clinical trials are necessary for testing the efficacy of the multidisciplinary therapeutic regimen.

## Figures and Tables

**Table 1:** Summary of studies in which the presence of *H. pylori* in dental plaque was determined by rapid urease test/CLO test.

No.	Author(s)	Year	Sample size	Prevalence of *H. pylori *
1	Assumpção et al. [48]	2010	99 adult patients who underwent upper gastrointestinal endoscopy	52%
2	Al Asqah et al. [35]	2009	Sixty-two dyspeptic patients with periodontitis and 39 dyspeptic patients without periodontitis	Overall-65%; 79% in periodontitis group and 43% in nonperiodontitis group
3	Anand et al. [38]	2006	Sixty-five dyspeptic patients with *H. pylori* infection (cases) and 69 dyspeptic patients without *H. pylori * infection (control)	Overall-79.9%; 89% among cases and 71% among controls
4	Chitsazi et al. [23]	2006	88 dyspeptic patients-44 with *H. pylori* infection and 44 without *H. Pylori* infection	Overall 18.2%; 36.4% in HP positive group
5	Choudhury et al. [22]	2003	124 patients with dyspepsia	43%
6	Gürbüz et al. [30]	2003	75 dyspeptic patients	91.7%
7	Suk et al. [45]	2002	Sixty-five patients with dyspeptic symptoms	100%
8	Avcu et al. [21]	2001	241 *H. pylori* positive patients with gastric histologic changes	44.8%
9	Özdemir et al. [49]	2001	81 dyspeptic patients	79%
10	Qureshi et al. [50]	1999	60 dyspeptic patients	50%
11	Contractor et al. [51]	1998	100 healthy subjects	81%

**Table 2:** Summary of studies in which the presence of *H. pylori* in dental plaque was determined by PCR.

No.	Authors	Year	Target gene	Sample size	Prevalence of *H. pylori *
1	Momtaz et al. [52]	2012	ureC, cagA, and vacA	300 patients with gastroduodenal diseases	None of the plaque samples showed presence of *H. pylori *
2	Agarwal and Jithendra [31]	2012	16S rRNA	30 *H. pylori* positive and 20 *H. pylori* negative patients	Overall-42%; in *H. pylori* positive group-60%; in *H. pylori* negative group-15%.
3	Bago et al. [41]	2011	16S rDNA	56 patients with chronic periodontitis and gastric *H. pylori* positive	37.5%
4	Chaudhry et al. [53]	2011	16srRNA and 860 bp DNA region	89 dyspeptic patients reporting for endoscopy	51.6% for both genes; 62.9% for 16srRNA; 61% for 860 bp DNA region and 73% if either of the 2 regions are considered
5	Gao et al. [44]	2011	ureC and cagA genes	96 patients with *H. pylori* infection	82.3%
6	Wichelhaus et al. [54]	2011	860bp DNA	11 orthodontic patients	36%
7	Assumpção et al. [48]	2010	vacA and cagA	99 adult patients who underwent upper gastrointestinal endoscopy	72% samples were positive for *H. pylori*. 63 of 71 positive dental plaque samples were positive for vacA and cagA. 58/71 were positive for cagA while vacA genotypes had a prevalence ranging from 13–59%
8	Rasmussen et al. [55]	2010	Genomic DNA	78 dyspeptic patients	47.4%
9	Eskandari et al. [56]	2010	16S rRNA	67 patients with chronic periodontitis-23 with *H. pylori* positive gastritis	5.97%
10	Silva et al. [57]	2010	vacA and 16S rDNA	30 dyspeptic patients	20% by 16S rDNA and 6.7% by vacA
11	Silva et al. [58]	2010	16S rRNA	115 patients	11.3%
12	Silva et al. [59]	2009	16s ribosomal and cagA genes	32 with *H. pylori* positive with gastric disease and 32 with *H. pylori *positive with no gastric disease	Overall-17.7%. Among cases, *H. pylori* DNA detected in 36.6% and cagA gene detected in 3 out of 11 (27.3%) samples. In control group 0%
13	Gonçalves et al. [60]	2009	JW22 and JW23 primers/16S rRNA	23, HIV seropositive individuals of whom 13 with chronic periodontitis and 10 with periodontally healthy and 31 HIV seronegative individuals of whom 17 had chronic periodontitis and 14 were periodontally healthy	Not specified
14	Liu et al. [61]	2009	860 bp fragment	443 dyspeptic patients	59.4%
15	Bürgers et al. [36]	2008	16S rDNA	94 patients who underwent upper gastrointestinal endoscopy	5.4%
16	Liu et al. [62]	2008	860 bp fragment	214 children	58.9%
17	Teoman et al. [25]	2007	Urease A	67 dyspeptic patients	28.3%
18	Olivier et al. [63]	2006	urease AB gene; phosphoglucosamine mutase (*glmM*) gene; and 860 bp DNA region	74 healthy members of a rural community	0
19	Kignel et al. [64]	2005	16S rRNA	49 dyspeptic patients	2%
20	Fritscher et al. [65]	2004	860 bp fragment	53 patients with recurrent aphthous stomatitis and 52 patients without RAS	Overall-3.8%; 5.7% in cases and 1.9% among controls
21	Gebara et al. [66]	2004	16S rDNA	30 dentate patients with gingivitis/periodontitis and *H. pylori* infection	20% in supra-gingival plaque and 26.6% in subgingival plaque
22	Umeda et al. [37]	2003	16S rRNA	56 dental patients	25%
23	Goosen et al. [67]	2002			
24	Berroteran et al. [39]	2002	Urease genes	32 dyspeptic patients and 20 asymptomatic controls	Overall-28.9%; 37.5% among dyspeptic patients and 15% among controls
25	Suk et al. [45]	2002	cagA	Sixty-five patients with dyspeptic symptoms	43.1%
26	Miyabayashi et al. [43]	2000	ureA	47 patients with chronic gastritis or peptic ulcer	38.3%
27	Song et al. [68]	2000	860 bp fragment	Forty-two patients who underwent upper gastrointestinal endoscopy	Overall 97% (82% in molar region, 64% in premolar region and 59% in incisor region)
28	Song et al. [69]	2000	860 bp fragment	20 dyspeptic patients	Not specified
29	Song et al. [70]	2000	860 bp fragment	21 patients	100%
30	Song et al. [71]	1999	Urease A, 16S rRNA, and 860 bp fragment	40 dental patients	
31	Dowsett et al. [18]	1999			Not specified
32	Oshowo et al. [32]	1998	16S rRNA	208 dyspeptic patients-116 *H. pylori* positive and 92 *H. pylori* negative	Overall 6.25% all in *H. pylori* positive
33	Hardo et al. [24]	1995	16S rRNA	62 dyspeptic patients	1.6%
34	Mapstone et al. [72]	1993	16S rRNA	21 dyspeptic patients-13 with *H. pylori* associated gastritis and 8 who had normal histology	Overall-9.5%; 15.4% in gastritis group and 0 in histologically normal group-overall prevalence-9.5%
35	Nguyen et al. [73]	1993	16S rRNA	25 dyspeptic patients	Overall 28% all in *H. pylori* positive; among *H. pylori* positive individuals 38.8%.

**Table 3:** Summary of studies in which the presence of *H. pylori* in dental plaque was determined by culture.

No.	Authors	Year	Sample size	Prevalence of *H. pylori *
1	Agarwal and Jithendra [31]	2012	30 *H. pylori* positive and 20 *H. pylori* negative patients	Overall-18%; in *H. pylori* positive group-30%; in *H. pylori* negative group-0
2	Loster et al. [74]	2009	Forty six dentists without known co-morbidities	48%
3	Teoman et al. [25]	2007	67 dyspeptic patients	0
4	Czesnikiewicz-Guzik et al. [75]	2005	100 female patients	48.3%
5	Cze$\overset{´}{\text{s}}$nikiewicz-Guzik et al. [33]	2004	100 female patients	48.3%
6	Umeda et al. [37]	2003	18 dental patients	5.6%
7	Goosen et al. [67]	2002	58 clinically healthy volunteers	13.8% of which only 5.2% were positive in PCR analysis
8	Checchi et al. [28]	2000	35 patients from a Periodontology clinic	8.6%
9	Oshowo et al. [32]	1998	208 dyspeptic patients-116 *H. pylori* positive and 92 *H. pylori* negative	Overall 1% all in *H. pylori* positive
10	Hardo et al. [24]	1995	62 dyspeptic patients	0
11	Krajden et al. [16]	1989	71 patients undergoing endoscopy	1.4%

**Table 4:** Summary of studies in which the presence of *H. pylori* in dental plaque was determined by EIA.

No.	Authors	Year	Sample size	Prevalence of *H. pylori *
1	Namiot et al. [26]	2010	155 patients	65.6%
2	Leszczyńska et al. [27]	2009	164 dyspeptic patients referred for endoscopy-95 *H. pylori* infected and 69 noninfected	82.1% in *H. pylori* positive subjects and 17.7% in *H. pylori* negative subjects
3	Checchi et al. [28]	2000	35 patients from a Periodontology clinic	11%
4	Savoldi et al. [29]	1998	80 dyspeptic patients	0

**Table 5:** Data regarding the coinfection of *H*. *pylori* and oral infection.

No.	Authors	Year	Method used to detect *H. pylori *	Sample size	Prevalence of coinfection of *H. pylori *
1	Agarwal and Jithendra [31]	2012	PCR-16S rRNA	30 *H*. *pylori* positive and 20 *H*. *pylori* negative patients	Overall-36%; in *H. pylori* positive group-60%
2	Agarwal and Jithendra [31]	2012	Culture	30 *H. pylori* positive and 20 *H. pylori* patients	Overall-18%; in *H. pylori* positive group-30%
3	Bago et al. [41]	2011	PCR-16S rDNA	56 patients with chronic periodontitis and gastric *H. pylori* positive	37.5%
4	Silva et al. [58]	2010	PCR-16S rRNA	115 patients	Overall-8.7%; among *H. pylori* positive group-14.93%
5	Eskandari et al. [56]	2010	PCR-16S rRNA	67 patients with chronic periodontitis-23 with *H. pylori* positive gastritis	Overall-5.97%; among *H. pylori* positive group-17.39%
6	Al Asqah et al. [35]	2009	RUT	62 dyspeptic patients with periodontitis and 39 dyspeptic patients without periodontitis	Overall-32.7%; 66% among *H. pylori* positive patients
7	Liu et al. [61]	2009	PCR-860-bp fragment	443 dyspeptic patients	Overall-42.7%; among *H. pylori* positive subjects-69.2% (75.5% in 18–29 years; 61.7% in 30–39 years; 79.7% in 40–49 years; 76.6% in ≥50 years)
8	Leszczyńska et al. [27]	2009	EIA	164 dyspeptic patients referred for endoscopy-95 *H. pylori* positive and 69 noninfected	Overall-47.6%; 82.1% in *H. pylori* positive
9	Silva et al. [59]	2009	PCR-16s ribosomal and cagA genes	30 with *H. pylori *positive with gastric disease and 32 with *H. pylori *positive with no gastric disease	Overall-17.7%. Among cases, *H. pylori* DNA detected in 36.6%, and cagA gene detected in 3 out of 11 samples. In control group-0%
10	Bürgers et al. [36]	2008	PCR-16S rDNA	94 patients who underwent upper gastrointestinal endoscopy	Overall-1.1% 3.5% of *H. pylori* positive subjects
11	Teoman et al. [25]	2007	PCR-Urease A	67 dyspeptic patients	Overall-25.4%; among *H. pylori* positive group-36.2%
12	Anand et al. [38]	2006	RUT	Sixty-five dyspeptic patients with *H. pylori* infection and 69 dyspeptic patients without *H. pylori *infection	Overall-43.3%; 89.2% among cases
13	Chitsazi et al. [23]	2006	RUT	88 dyspeptic patients-44 with *H. pylori* infection and 44 without *H. pylori* infection	Overall 34.1%; 36.4% in *H. pylori* positive group
14	Agüloğlu et al. [76]	2006	CLO test	468 patients who were *H. pylori* positive by CLO test	25.2%
15	Agüloğlu et al. [76]	2006	EIA	318 patients who were *H. pylori* positive by EIA	23.6%
16	Agüloğlu et al. [76]	2006	Culture	295 patients who were *H. pylori* positive by culture	14.6%
17	Czesnikiewicz-Guzik et al. [75]	2005	Culture	100 female patients	6.9%
18	Kignel et al. [64]	2005	PCR-16S rRNA	49 dyspeptic patients	2% of the total population and 5% of *H. pylori* positive subjects
19	Umeda et al. [37]	2003	PCR-16S rRNA	56 dental patients	Overall-14.3%; among *H. pylori* positive group-28.6%.
20	Gürbüz et al. [30]	2003	RUT	75 dyspeptic patients	Overall-81.3%; among *H. pylori* positive subjects-93.9%
21	Berroteran et al. [39]	2002	PCR-Urease genes	32 dyspeptic patients and 20 asymptomatic controls	Overall-13.5%; 58% among dyspeptic patients
22	Suk et al. [45]	2002	PCR-cagA	65 patients with dyspeptic symptoms	Overall-43.1%, 73.7% among *H. pylori* positive patients
23	Suk et al. [45]	2002	RUT	65 patients with dyspeptic symptoms	Overall-58.5%, 100% among *H. pylori* positive patients
24	Özdemir et al. [49]	2001	CLO test	81 dyspeptic patients	Overall-64.2%; among *H. pylori* positive group-82.5%.
25	Song et al. [70]	2000	PCR-860-bp fragment	21 patients	Overall-47.6%; among *H. pylori* positive group-100%
26	Qureshi et al. [50]	1999	CLO test	60 dyspeptic patients	Overall 33.3%; in *H. pylori* positive 55.6%
27	Oshowo et al. [32]	1998	PCR-16S rRNA and culture	208 dyspeptic patients-116 *H. pylori* positive and 92 *H. pylori* negative	By PCR-Overall 6.25%; in *H. pylori* positive-11.2% By culture-Overall 1%; in *H. pylori* positive-1.7% By both methods-Overall 7.2% in *H. pylori* positive-12.9%.
28	Hardo et al. [24]	1995	16S rRNA	62 dyspeptic patients	0
29	Mapstone et al. [72]	1993	PCR-16S rRNA	21 dyspeptic patients-13 with *H. pylori* associated gastritis and 8 who had normal histology	15.4% in gastritis group-overall prevalence 9.5%
30	Nguyen et al. [73]	1993	PCR-16S rRNA	25 dyspeptic patients	Overall 28%, among *H. pylori* positive individuals 38.8%
31	Krajden et al. [16]	1989	Culture	71 patients undergoing endoscopy	1.4% of the total population and 3.5% of *H. pylori* positive subjects

**Table 6:** Data regarding the association between periodontal diseases and *H*. *pylori* infection.

No.	Authors	Year	Definition of gingival/periodontal disease	Sample size	Association with oral *H. pylori *	Association with gastric *H. pylori *
1	Silva et al. [58]	2010	At least 4 teeth with PD ≥ 5 mm and CAL > 3 mm	115 dyspeptic patients	Significant	Not evaluated
2	Namiot et al. [26]	2010	Russell's periodontal index	155 dyspeptic patients	Nonsignificant	Not evaluated
3	Gonçalves et al. [60]	2009	At least 3 sites with PD ≥ 5 mm and/or CAL ≥ 4 mm and BOP	23 HIV seropositive patients of whom 13 had periodontitis and 10 were periodontally healthy; 31 HIV seronegative patients of 17 had periodontitis and 14 were periodontally healthy	Significant	
4	Al Asqah et al. [35]	2009	BOP + PD ≥ 3 mm on at least 4 teeth	Dyspeptic patients-62 patients with periodontitis and 39 without periodontitis	Significant	Significant
5	Liu et al. [61]	2009	Gingival index	443 dyspeptic patients	Significant	Not evaluated
6	Zaric et al. [40]	2009	Mean PD, CAL, and gingival index scores	66 dyspeptic patients with *H. pylori* infection of gastric mucosa	Significant for mean PD and CAL; not significant for gingival index scores	Not evaluated
7	Bürgers et al. [36]	2008	Periodontal Screening Index	94 dyspeptic patients	Nonsignificant	Nonsignificant
8	Namiot et al. [77]	2007	Russell's periodontal index	137 *H. pylori* positive patients with peptic ulcer	Outcome variable was efficacy of HP eradication	Nonsignificant (outcome variable was efficacy of *H. pylori* eradication)
9	Anand et al. [38]	2006	Patients with one or more sites with a PD ≥ 3 mm and CAL ≥ 3 mm at the same site	Sixty-five dyspeptic patients with *H. Pylori* infection and 69 dyspeptic patients without *H. pylori* infection	Not evaluated	Nonsignificant
10	Gebara et al. [66]	2004	Gingivitis group-patients with PD ≤ 3 mm and BOP on at least 4 sites; periodontitis group-BOP + PD ≥ 5 mm on at least 4 teeth	15 gingivitis and 15 periodontitis patients-All were *H. pylori* positive in antral mucosa	Nonsignificant	
11	Umeda et al. [37]	2003	Presence of periodontal pockets ≥ 4 mm	28 patients who harbored *H. pylori* in stomach/duodenum	Significant	Not evaluated
12	Choudhury et al. [22]	2003	CPI	124 dyspeptic patients	Significant	Not evaluated
13	Dye et al. [34]	2002	Presence of 1 dental site with PD ≥ 5 mm	Data from 4504 participants of NHANES III Survey	Not evaluated	Significant
14	Berroteran et al. [39]	2002	Gingival index-scoring 0–3	32 dyspeptic patients and 20 asymptomatic controls	Nonsignificant	Nonsignificant
15	Dowsett et al. [18]	1999	Full mouth periodontal examination	242 subjects	Nonsignificant	Nonsignificant
16	Savoldi et al. [29]	1998	Gingival index	80 dyspeptic patients	Nonsignificant-None of the plaque samples were positive for HP	Nonsignificant
17	Hardo et al. [24]	1995	CPITN	62 dyspeptic patients	Nonsignificant	Nonsignificant
18	Nguyen et al. [73]	1993	Gingival index	25 dyspeptic patients	Nonsignificant	Nonsignificant

**Table 7:** Data regarding effects of anti-*H*. *pylori* therapy on dental plaque.

No.	Authors	Year	Method used to detect *H. pylori *	Sample size	Prevalence of *H. pylori *	Prevalence after anti-*H. pylori* therapy	Effect on *H. pylori* infection
1	Gao et al. [44]	2011	PCR-ureC and cagA genes	80 patients with *H. pylori* infection-37 treated with anti-*H. pylori* therapy and 43 treated with anti-*H. pylori* therapy and periodontal therapy		After 4 weeks-29.7% in gp A and 4.7% in gp B; after 1 year-43.2% in gp A and 18.6% in gp B	Eradication rate of gastric *H. pylori*. After 4 weeks-73% in gp A and 81.4% in gp B After 1 year-32.4% in gp A and 62.8% in gp B
2	Bago et al. [41]	2011	PCR-16S rDNA	56 patients with chronic periodontitis and gastric *H. pylori* positive	37.5% (*n* = 21)	0	Eradication rate in stomach was 76.2%
3	Zaric et al. [40]	2009	PCR	44 patients: 21 patients positive for *H. pylori* in subgingival dental plaque and gastric mucosa and 23 patients who were positive for *H. pylori* only in gastric mucosa-all 44 received only for *H. pylori* (triple) therapy		In G^+^O^+^t-66.7%	In the G^+^O^+^t group, only 47.6% showed eradication of gastric *H. pylori* compared to 87.4% in G^+^O^−^t
4	Gebara et al. [42]	2006	PCR-16S rDNA	30 dentate patients with gingivitis/periodontitis and *H. pylori* infection who received anti-*H. pylori* therapy	20% (*n* = 6) in supra-gingival plaque and 26.6% (*n* = 8) in sub-gingival plaque	30% in supra-gingival plaque and 46.7% in sub-gingival plaque	
5	Gürbüz et al. [30]	2003	CLO test	75 dyspeptic patients of which 61 were *H. pylori* positive and also had *H. pylori* in dental plaque	90.7% (*n* = 68); 81.3% (*n* = 61) had co-infection	100%	
6	Suk et al. [45]	2002	PCR-cagA	Sixty-five patients with dyspeptic symptoms	Overall-43.1% (*n* = 28), 73.7% (28/38) among *H. pylori* positive patients	92.9%	
7	Butt et al. [46]	2001	Smear cytology	82 patients positive for *H. pylori* in dental plaque: 27 received only anti-*H. pylori* therapy (gp 1); 25 received anti-HP therapy + periodontal therapy (gp 2); 30 received only periodontal therapy (gp 3)	100%	100% in gp 1; 16% in gp 2; 10% in gp 3	
8	Miyabayashi et al. [43]	2000	PCR-ureA	47 patients with chronic gastritis or peptic ulcer-48.9% (*n* = 23) were positive for oral HP and 38.3% (*n* = 18) had HP in plaque		Oral prevalence at 4 weeks-31.9	At 4 weeks-91.6% of subjects negative for oral *H. pylori* were successfully eradicated of HP infection compared to 52.2% in oral *H. pylori* positive patients. At 2 years-95.8% of subjects negative for oral *H. pylori* were successfully eradicated of *H. pylori* infection compared to 69.5% in oral *H. pylori* positive patients

**Table 8:** Data regarding effects of periodontal therapy on *H*. *pylori* in dental plaque and gastric infection.

No.	Authors	Year	Method used to detect *H. pylori *	Sample size	Prevalence of *H. pylori *	Prevalence after anti-*H. pylori* therapy	Effect on *H. pylori* infection
1	Gao et al. [44]	2011	PCR-ureC and cagA genes	80 patients with *H. pylori* infection-37 treated with anti-HP therapy (gp A) and 43 treated with anti-*H. pylori* therapy and periodontal therapy (gp B)		After 4 weeks-29.7% in gp A and 4.7% in gp B; after 1 year-43.2% in gp A and 18.6% in gp B	Eradication rate of gastric *H. pylori.* After 4 weeks-73% in gp A and 81.4% in gp B, After 1 year-32.4% in gp A and 62.8% in gp B
2	Zaric et al. [40]	2009	PCR	43 patients positive for *H. pylori* in sub gingival dental plaque and gastric mucosa: 21 received only anti-*H. pylori* triple therapy (G^+^O^+^t); 22 received anti-*H. pylori* triple therapy + periodontal therapy (G^+^O^+^tp)		In G^+^O^+^t-66.7%; in G^+^O^+^tp-27.3%	In the G^+^O^+^tp group, 77.3% showed eradication of gastric *H. pylori* compared to 47.6% in G^+^O^+^t. *H. pylori *eradication in the stomach and the oral cavity coincided—that is, all 16 of the individuals negative for oral *H. pylori *were also negative for gastric *H. pylori*. Five of the participants positive for oral samples were positive for gastric *H. pylori *as well.
3	Jia et al. [47]	2009		107 dyspeptic patients-56 received dental plaque control (test) and 51 did not (control)			Prevalence of *H. pylori* in gastric mucosa was 19.64% in test group and 84.31% in control group
4	Butt et al. [46]	2001	Smear cytology	82 patients positive for *H. pylori* in dental plaque: 27 received only anti-*H. pylori* therapy (gp 1); 25 received anti-*H. pylori* therapy + periodontal therapy (gp 2); 30 received only periodontal therapy (gp 3)	100%	100% in gp 1; 16% in gp 2; 10% in gp 3	
